# Prevalence of SARS-CoV-2 Infections Among Students, Teachers, and Household Members During Lockdown and Split Classes in Berlin, Germany

**DOI:** 10.1001/jamanetworkopen.2021.27168

**Published:** 2021-09-28

**Authors:** Welmoed van Loon, Stefanie Theuring, Franziska Hommes, Marcus A. Mall, Joachim Seybold, Tobias Kurth, Frank P. Mockenhaupt

**Affiliations:** 1 Institute of Tropical Medicine and International Health, Charité–Universitätsmedizin Berlin, corporate member of Freie Universität Berlin and Humboldt-Universität zu Berlin, Berlin, Germany; 2 Department of Pediatric Respiratory Medicine, Immunology, and Critical Care Medicine, Charité–Universitätsmedizin Berlin, corporate member of Freie Universität Berlin and Humboldt-Universität zu Berlin, Berlin, Germany; 3 Medical Directorate, Charité–Universitätsmedizin Berlin, corporate member of Freie Universität Berlin and Humboldt-Universität zu Berlin, Berlin, Germany; 4 Institute of Public Health, Charité–Universitätsmedizin Berlin, corporate member of Freie Universität Berlin and Humboldt-Universität zu Berlin, Berlin, Germany

## Abstract

This cohort study assesses the prevalence of SARS-CoV-2 among students, teachers, and household members during lockdown and split classes in 2021 in Berlin, Germany, and examines the association between pandemic restrictions and student health-related quality of life.

## Introduction

Children tend to bear a smaller proportion of the COVID-19 disease burden but are particularly affected by pandemic restrictions, including school closures.^1^ The occurrence of SARS-CoV-2 infection in school communities tends to be isolated and to produce few secondary cases.^2,3^ Between June 2020 and March 2021, we examined 24 school classes (12 primary and 12 secondary) across Berlin, Germany, on 4 occasions. In November 2020, there were 9 (2.7%), 2 (1.4%), and 14 (2.3%) SARS-CoV-2 infections among 338 students, 140 teachers, and 611 household members during the second pandemic peak, respectively (7-day incidence of 185 to 210 per 100,000). No secondary cases occurred among individuals in classes.^3^ After SARS-CoV-2 infections declined in early 2021, they increased again in mid-February and peaked by mid-April (Figure). In parallel, the SARS-CoV-2 B.1.1.7 variant gained predominance.^4^ Here, we present data observed with our cohort (1) at the end of February 2021, after a 2-month lockdown, and (2) at the end of March 2021, 2 to 3 weeks after schools resumed instruction with split classes half of the original size attending school on alternate weeks.

**Figure.**
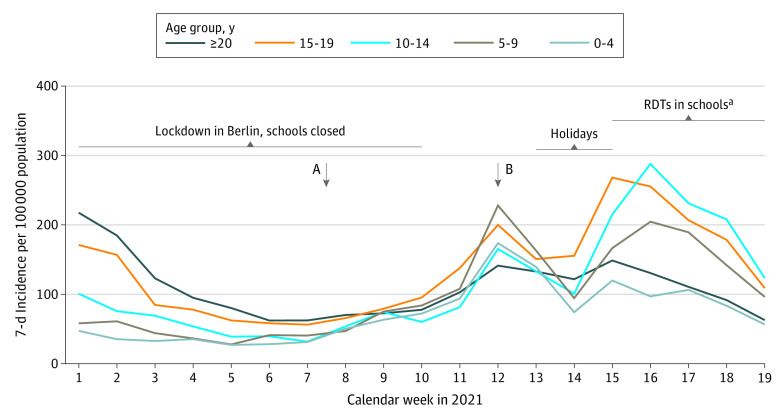
Age-Stratified SARS-CoV-2 School Community Incidence in Berlin, Germany, in February and March 2021 Arrows indicate the time points of the cross-sectional assessment during lockdown (A, mid-February 2021) and split-class schooling (B, end of March 2021). Data were derived from the Berlin State Office for Health and Social Affairs.^6^ ^a^Twice-weekly mandatory testing in schools with rapid diagnostic tests (RDTs).

## Methods

The details of this study were described previously.^3^ Classes comprising 327 students, 142 staff members, and 591 associated household members were tested for SARS-CoV-2 infection during lockdown between February 19 and 26, 2021. Split classes comprising 324 students, 133 staff members, and 591 associated household members were tested on March 25, 2021. Mandatory infection prevention and control measures for schools included an absence rule for symptomatic individuals, frequent ventilation, 1.5-m distancing, and face mask wearing. Rapid diagnostic tests (from Roche, Siemens, and Nal von Minden, among others) were distributed to schools by the Senate of Berlin for voluntary use. Illustrated self-swabbing kits (oropharynx, both nostrils; CoronaOne) were sent to participants and collected within 24 hours. Reverse transcriptase–polymerase chain reaction (GFE Blut) was used to determine SARS-CoV-2 infection. Participant symptoms and behavior were documented. In March 2021, we measured students’ health-related quality of life (HRQOL) by administering the KIDSCREEN-10 questionnaire for children. Individual scores were transformed into T scores. Low HRQOL was defined as 0.5 SD below the mean T score of (prepandemic) reference data for children.^5^

This study followed the Strengthening the Reporting of Observational Studies in Epidemiology (STROBE) reporting guideline. Informed written consent and assent was obtained from all participants and their legal guardians in the case of minors. The Charité–Universitätsmedizin Berlin ethics committee approved the study.

## Results

We obtained swabs from 1044 of 1060 participants (98.5%) during lockdown and from 898 of 1048 participants (85.7%) during split classes. In the latter period, 271 of 309 students (87.7%) and 101 of 123 staff members (82.1%) had attended school at least once in the preceding 14 days, with a median of 6 and 9 days (range, 1-11), respectively. Further characteristics are shown in the Table.

**Table.** Selected Characteristics of Students, School Staff Members, and Household Members in Berlin, Germany, in February and March 2021

Characteristic	No./Total (%)		
	Students	School staff members	Household members
**February 2021 (lockdown)**			
Participants	327	142	591
Sex			
Male	167	38	293
Female	160	104	296
Age, median (range), y	15.0 (9.0-19.0)	47.0 (28.0-65.0)	42.0 (2.0-86.0)
SARS-CoV-2 positive test result	0/324	0/138	1/582 (0.2)
Moderate to very high fear of SARS-CoV-2 infection^a^	67/170 (39.4)	78/118 (66.1)	157/323 (48.6)
Meeting friend(s) in the last 14 d			
Never	38/168 (22.6)	33/118 (28.0)	97/319 (30.4)
1-2 times/wk	96/168 (57.1)	79/118 (66.9)	190/319 (59.6)
Playing sports			
Never	25/169 (14.8)	30/118 (25.4)	84/322 (26.1)
1-2 times/wk	76/169 (45.0)	50/118 (42.4)	137/322 (42.5)
Frequent^b^			
Computer gaming	96/169 (56.8)	11/117 (9.4)	85/321 (26.5)
Television and/or video (YouTube) watching	150/169 (88.8)	79/115 (68.7)	232/319 (72.7)
**March 2021 (split classes)**			
Participants	324	133	591
Sex			
Male	163	37	283
Female	161	96	308
Age, median (range), y	15.0 (9.0-18.0)	47.0 (30.0-65.0)	42.0 (2.0-86.0)
SARS-CoV-2 positive test result	2/263 (0.8)	1/112 (0.9)	3/523 (0.6)
Moderate to very high fear of SARS-CoV-2 infection^a^	73/180 (40.6)	77/113 (68.1)	161/318 (50.6)
Meeting friend(s) in the last 14 d			
Never	42/179 (23.5)	39/106 (36.8)	113/316 (35.8)
1-2 times/wk	98/179 (54.7)	61/106 (57.5)	175/316 (55.4)
Playing sports			
Never	26/179 (14.5)	37/112 (33.0)	93/316 (29.4)
1-2 times/wk	77/179 (43.0)	49/112 (43.8)	128/316 (40.5)
Frequent^b^			
Computer gaming	96/179 (53.6)	12/112 (10.7)	87/318 (27.4)
Television and/or video (YouTube) watching	152/177 (85.9)	73/108 (67.6)	226/312 (72.4)
HRQOL score^c^			
Low	77/174 (44.3)	NA	NA
Within the normal range	63/174 (36.2)	NA	NA
High	34/174 (19.5)	NA	NA

Abbreviations: HRQOL, health-related quality of life; NA, not applicable.

^a^ Participants were asked, “How afraid are you of contracting the coronavirus?”

^b^ Daily or ≥3 days per week.

^c^ Individual raw scores were transformed into T scores. Low and high HRQOL scores were defined as 0.5 SD below and above the mean in (prepandemic) reference data for primary and secondary school-aged children in Europe, with mean (SD) values of 53.90 (10.73) and 48.51 (9.28) for our respective subgroups.^5^

Of 1044 participants who underwent testing during lockdown in February 2021, we detected 1 symptomatic adult household member with SARS-CoV-2 infection (0.1%; 95% CI, 0.0%-0.5%). Four weeks later in March 2021, 6 of 898 individuals with split classes had SARS-CoV-2 infection (0.7%; 95% CI, 0.2%-1.4%). Of these 6 individuals, 2 students and 3 household members had a known positive test result and/or were quarantined and 1 teacher was asymptomatic. The students and teacher with SARS-CoV-2 infection attended different schools.

In March 2021, 73 of 180 students (40.6%) expressed moderate to very strong fear of infection (in response to the question “How afraid are you of contracting the coronavirus?”), 77 of 174 (44.3%) exhibited a low HRQOL score, 140 of 179 (78.2%) never or rarely saw friends, and 103 of 179 (57.5%) never or rarely played sports in the preceding 14 days. Computer gaming (96 of 179; 53.6%) and television/video (YouTube) watching (152 of 177; 85.9%) were common among students (Table).

## Discussion

In early 2021, we detected only isolated SARS-CoV-2 infections, no clusters, and 1 school attendee with an infection. This low level of infection at schools confirms our previous data.^3^ Moreover, students’ decreased HRQOL scores and participation in leisure activities illustrate the deleterious outcomes associated with almost 1 year of pandemic restrictions. Ongoing work will further detail these outcomes. Berlin’s SARS-CoV-2 incidence pattern shows a rapid increase (Figure), particularly among children, 2 weeks postlockdown during split-class schooling in March 2021.^6^ Exponential growth in incidence began weeks earlier, although we cannot exclude an acceleration of SARS-CoV-2 infection as a result of school attendance. Other explanations include available rapid tests for students beginning in mid-March and increased SARS-CoV-2 transmission attributable to the B.1.1.7 variant. Limitations of our study include its small sample size, 2 cross-sectional time points, and voluntary participation potentially leading to selection bias. Our data support that school closures should be the last resort in controlling the SARS-CoV-2 pandemic.

## References

[1] Ravens-Sieberer U, Kaman A, Erhart M, Devine J, Schlack R, Otto C. Impact of the COVID-19 pandemic on quality of life and mental health in children and adolescents in Germany. Eur Child Adolesc Psychiatry. Published online January 25, 2021. doi:10.1007/s00787-021-01726-5 33492480 PMC7829493

[2] Ismail SA, Saliba V, Lopez Bernal J, Ramsay ME, Ladhani SN. SARS-CoV-2 infection and transmission in educational settings: a prospective, cross-sectional analysis of infection clusters and outbreaks in England. Lancet Infect Dis. 2021;21(3):344-353. doi:10.1016/S1473-3099(20)30882-3 33306981 PMC7833602

[3] Theuring S, Thielecke M, van Loon W, SARS-CoV-2 infection and transmission in school settings during the second COVID-19 wave: a cross-sectional study, Berlin, Germany, November 2020. Eurosurveillance. Accessed August 26, 2021. https://www.eurosurveillance.org/content/10.2807/1560-7917.ES.2021.26.34.2100184 10.2807/1560-7917.ES.2021.26.34.2100184 PMC8393892 34448448

[4] van Loon W, Rössig H, Burock S, Emergence of SARS-CoV-2 B.1.1.7 lineage at outpatient testing site, Berlin, Germany, January-March 2021. Emerg Infect Dis. 2021;27(7):1931-1934. doi:10.3201/eid2707.210845 34152970 PMC8237906

[5] KIDSCREEN Group Europe. *The KIDSCREEN Questionnaires—Quality of Life Questionnaires for Children and Adolescents. Handbook*. Pabst Science Publishers; 2006. Accessed May 28, 2021. https://s2f1ad284f5ffc52e.jimcontent.com/download/version/1494315548/module/11487374212/name/KIDSCREEN_manual_English.pdf

[6] Berlin State Office for Health and Social Affairs. COVID-19 in Berlin, distribution by age group. Published April 5, 2020. Accessed May 20, 2021. https://www.berlin.de/lageso/gesundheit/infektionskrankheiten/corona/tabelle-altersgruppen-gesamtuebersicht/

